# Comparative Histological Analysis of Dentine-Derived Tooth Grafts in Maxillary vs Mandibular Socket Preservation: A Retrospective Study of 178 Cases

**DOI:** 10.3390/dj12100320

**Published:** 2024-10-07

**Authors:** Elio Minetti, Francesco Gianfreda, Patrizio Bollero, Ciro Annicchiarico, Monica Daniele, Rossella Padula, Filiberto Mastrangelo

**Affiliations:** 1 Department of Biomedical, Surgical, Dental Science, University of Milan, 20161 Milan, Italy; elio.minetti@gmail.com; 2 Department of System Medicine, University of Rome “Tor Vergata”, 00133 Rome, Italy; patrizio.bollero@ptvonline.it; 3 Independent Researcher, 70100 Bari, Italy; 4 Faculty of Medicine and Surgery, University of Foggia, 71100 Foggia, Italy; monica.daniele@students.unifg.it (M.D.); rossella.pedula@students.unifg.it (R.P.); 5 Department of Clinical and Experimental Medicine, University of Foggia, 71100 Foggia, Italy

**Keywords:** alveolar ridge preservation, tooth-derived materials, demineralized dentin, Tooth Transformer^®^, socket preservation, histomorphometric analysis, bone regeneration, post-extractive sockets, bone tissue reconstruction

## Abstract

(1) Background: In recent years, there has been a growing interest in tooth-derived materials as valuable alternatives to synthetic biomaterials for preventing alveolar ridge dimensional changes. This study aimed to evaluate the histological and clinical differences between alveolar ridge preservation procedures in the maxilla and mandible using demineralized dentin treated with Tooth Transformer^®^. (2) Methods: A total of 178 patients in good general health were enrolled, with 187 post-extractive sockets lacking buccal and/or palatal bone walls. Alveolar socket preservation procedures and histological evaluations were performed. The sites were divided into two groups: Group A (99 mandibular samples) and Group B (108 maxillary samples). After 5 months (±1 month), single bone biopsies were performed for histologic and histomorphometric analysis. (3) Results: Clinical outcomes demonstrated a good healing of hard and soft tissues with an effective maintenance of bone architecture in both groups. Histomorphometric analysis revealed a total bone volume of 50.33% (±14.86) in Group A compared to 43.53% (±12.73) in Group B. The vital new bone volume was 40.59% (±19.90) in Group A versus 29.70% (±17.68) in Group B, with residual graft dentin material volume at 7.95% (±9.85) in Group A compared to 6.75% (±9.62) in Group B. (4) Conclusions: These results indicate that tooth-derived material supports hard tissue reconstruction by following the structure of the surrounding bone tissue. A 6.8% difference observed between the maxilla and mandible reflects the inherent disparities in natural bone structures in these regions. This suggests that the bone regeneration process after tooth extraction adheres to an anatomical functional pattern that reflects the specific bone characteristics of each area, thus contributing to the preservation of the morphology and functionality of the surrounding bone tissue.

## 1. Introduction

Tooth extraction invariably leads to changes in both the hard and soft tissues of the alveolar ridge. These tissue changes are critical for promoting healing, the closure of the socket, and restoring tissue homeostasis. Araujo and Lindhe (2005) [1] demonstrated that in the eight weeks following tooth extraction, there is a significant increase in osteoclastic activity, leading to substantial alterations in the walls of the post-extraction socket, with greater resorption observed on the buccal aspect compared to the lingual aspect. This results in both vertical and horizontal bone loss.

The irreversible process of three-dimensional alveolar bone resorption averages approximately 1.67 mm in vertical height and 3.87 mm in volume within one year post-extraction [2,3]. The surgical approaches for post-extractive socket preservation vary depending on the residual anatomy of the defect. Elian et al. [4] classified these defects into three types, with Chu et al. [5] further subclassifying type 2 extraction sockets based on precise bone dehiscence measurements.

The use of tooth-derived materials as grafts, introduced over 50 years ago by Yeomans and Urist, demonstrated the osteoinductive potential of demineralized dentin matrix [6,7,8]. Recent studies have extensively explored tooth graft regeneration, including the use of a novel medical device (Tooth Transformer SRL, Milan, Italy), which facilitates the creation of suitable tooth graft materials from a patient’s extracted tooth [9,10,11].

Autogenous tooth grafts have shown promising results in guided bone regeneration, with studies indicating their potential to support new bone formation and maintain alveolar ridge dimensions. The biological properties of dentin, including its composition and the presence of growth factors, contribute to its effectiveness as a bone graft material [12].

To compare the role of tooth-derived grafts with other graft materials such as allogeneic and synthetic bone, tooth-derived grafts offer unique advantages due to their dual osteoinductive and osteoconductive properties. Studies have shown that demineralized dentin matrix (DDM) derived from extracted teeth contains bone morphogenetic proteins (BMPs), which promote new bone formation—an ability typically associated with autologous bone grafts but often lacking in synthetic and allogeneic materials. For instance, Bessho et al. [13] found that BMPs in dentin support osteogenesis, making tooth-derived grafts comparable to autografts in terms of promoting bone regeneration [9].

Maxillary bone is less dense and more porous, with a higher proportion of trabecular bone, leading to increased vascularization and rapid healing but significant resorption post-extraction. Histologically, it shows larger marrow spaces and thinner trabeculae. In contrast, mandibular bone is denser with a higher proportion of cortical bone, providing greater mechanical strength and stability. It has thicker trabeculae and smaller marrow spaces, resulting in slower healing but long-term stability [14]. These differences necessitate tailored approaches for alveolar ridge preservation, with maxillary bone favoring quick graft integration and mandibular bone requiring materials that support sustained stability.

The aim of this retrospective study was to evaluate and compare the clinical and histological outcomes of alveolar ridge preservation procedures in the maxilla and mandible using demineralized dentin grafts processed using the Tooth Transformer^®^. By analyzing bone biopsies after a 5-month healing period, the study sought to investigate the differences in bone regeneration and graft integration between these two anatomical regions, providing insights into the effectiveness of tooth-derived grafts for enhancing bone preservation in both maxillary and mandibular sites.

## 2. Materials and Methods

### 2.1. Patient Selection

Patients were identified through the review of digital medical records and histological results, selecting all cases where the patient underwent socket preservation treatments with demineralized dentin from January 2017 to June 2024. Demographic information was collected. The extraction site defects were classified according to the criteria of Elian et al. [4], specifically focusing on class 2 defects. To accurately assess the dehiscence of the buccal bone at the extraction site, patients underwent a preliminary cone-beam computed tomography (CBCT) scan of the affected area prior to the day of surgery with the aim of evaluating whether the defect was suitable for socket preservation. In addition, the quantity of healthy root and dentine was verified for the preparation of the subsequent demineralized graft material.

All collected data were anonymized to ensure patient privacy and confidentiality. The study was conducted in accordance with the ethical principles outlined in the latest version of the Helsinki Declaration. Ethical approval for the clinical study protocol was granted by the University of Chieti Ethics Committee (Italy) on 21 March 2019 under the registration number 638-21/3/19.

### 2.2. Inclusion Criteria

The study included subjects who met the following criteria:

1. Subjects who underwent surgical intervention for tooth removal and alveolar socket preservation using exclusively a tooth-derived bone substitute (Tooth Transformer^®^—Tooth Transformer SRL).
2. Subjects with type 2 bone wall defects according to Elian et al. [4]’s classification, specifically subclass 2A as per Chu et al. [5]’s subclassification.
3. Subjects who did not have any systemic diseases or conditions that could impair bone metabolism.

### 2.3. Preoperative and Surgical Procedures

All patients received oral hygiene instructions and debridement two weeks prior to surgery. Before the intervention, patients rinsed with 0.2% chlorhexidine mouthwash for one minute (Curasept, Curaden Healthcare, Saronno, Italy). Prophylactic antibiotic therapy was administered, consisting of amoxicillin and clavulanic acid (1 g taken one hour before tooth extraction and 1 g three times daily for the next four days). Local anesthesia was achieved using articaine hydrochloride with epinephrine 1:100,000 (Orabloc, Pierrel, Milan, Italy).

Patients were divided into two groups based on the zone of surgery: Group A (mandibular) and Group B (maxillary). All extracted teeth were either molars or premolars, and regeneration surgery was performed immediately after tooth extraction and tooth transformation procedures, which typically lasted about 30 min. The vitality of the tooth (vital, non-vital, or undergoing endodontic root canal therapy) was not considered a differentiating factor, as previous studies demonstrated no significant differences when following the tooth transformation protocol.

**Figure 1:**
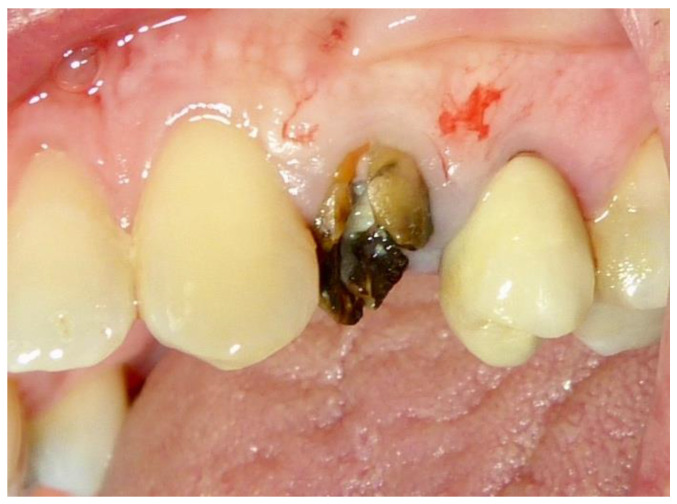
Tooth 2.4 before extraction.

**Figure 2:**
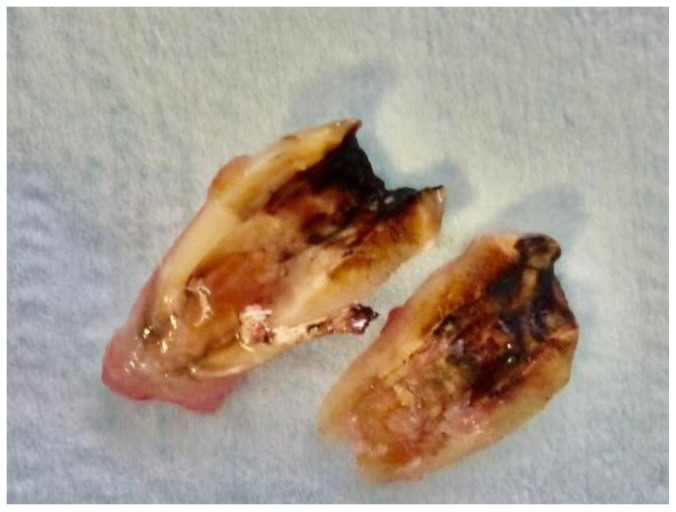
First upper premolar extracted. Decay was eliminated before TT treatment.

**Figure 3:**
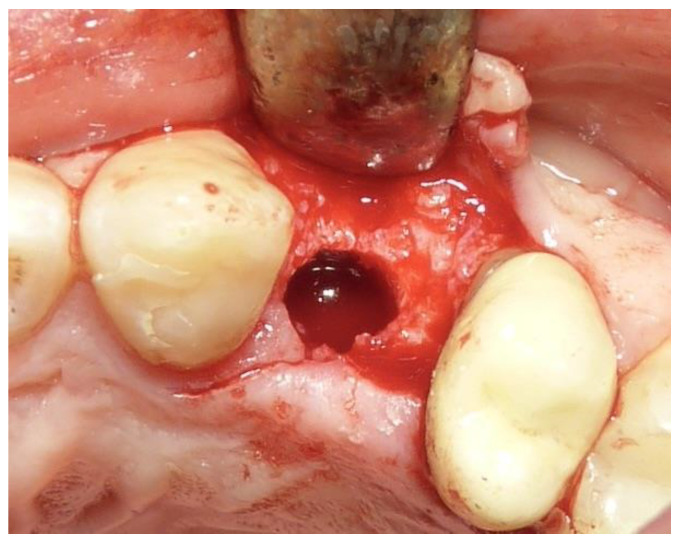
Post-extraction alveolus.

The extracted tooth was meticulously cleaned to remove any residual calculus and thoroughly polished using a diamond drill (ref. 6855—Dentsply Maillefer), with generous saline solution irrigation (Figure 1, Figure 2 and Figure 3). The procedure required the complete removal of any root filling material from the selected tooth. Subsequently, the tooth was finely cut into small pieces, and these fragments were placed in the mill (Tooth Transformer^®^, Tooth Transformer SRL, Milan, Italy), following the manufacturer’s guidelines. A single-use box containing 0.1 M hydrochloric acid, 10% hydrogen peroxide, and demineralized water was introduced for the transformation process.

**Figure 4:**
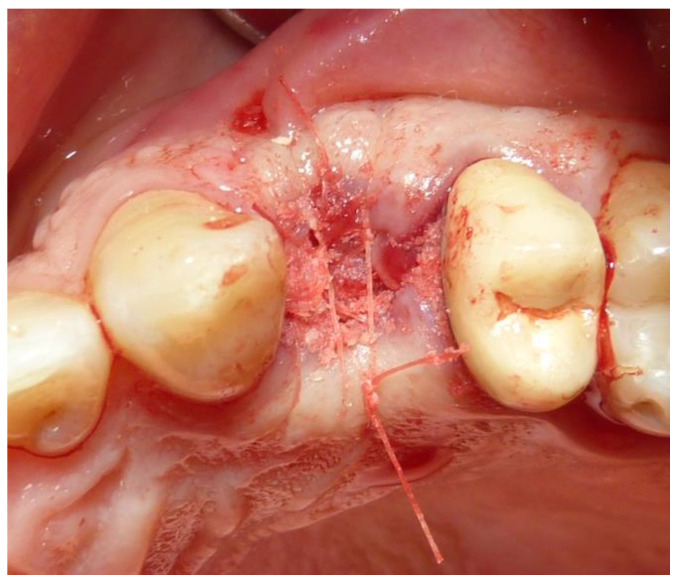
Particle graft biomaterials were deposited into the alveolar post-extractive sockets and covered with a resorbable collagen membrane (Osseoguard, Zimmer Biomet, Warsaw, IN, USA). Soft tissues were sutured with bioresorbable. (Vicryl 5-0 Ethicon Inc., Johnson & Johnson, Bridgewater).

After 25 min, the resulting particle graft biomaterials were deposited into the alveolar post-extractive sockets and covered with a resorbable collagen membrane (Osseoguard, Zimmer Biomet, Warsaw, IN, USA). The graft material was positioned into the sockets and covered with the membrane to achieve primary wound closure. Soft tissues were sutured with bioresorbable sutures (Vicryl 5-0, Ethicon Inc., Johnson & Johnson, Bridgewater, NJ, USA) (Figure 4).

Postoperative care included the administration of amoxicillin (2 g daily for 7 days, Pfizer, New York, NY, USA), and all sutures were removed after 14 days. Patients were instructed to rinse their mouths twice daily for one week with 0.2% chlorhexidine digluconate mouthwash and to take anti-inflammatory medication for pain management (nimesulide 100 mg twice daily). A soft diet was recommended for several days to avoid the mechanical contact of food with the surgical site. Sutures were removed seven days post-surgery.

**Figure 5:**
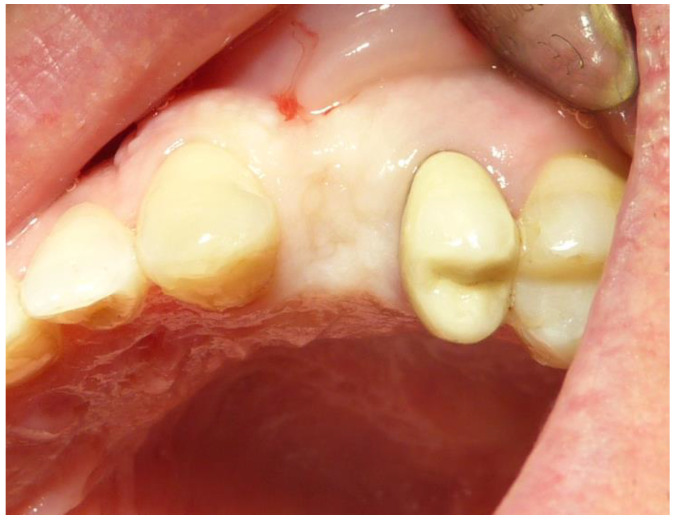
Post-surgical site healed after 5 months.

**Figure 6:**
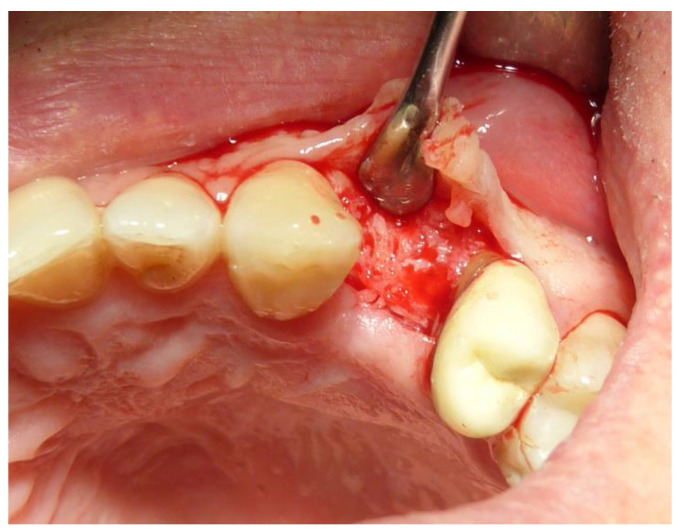
Regenerated bone visible after 5 months.

After a 5-month period (±1 month), implant placement was carried out (Figure 5 and Figure 6). For the preparation of the implant site, a 3 mm trephine cylindrical bur (3 × 18 mm, Meisinger, Neuss, Germany) was used under saline solution to collect a histological sample. A standard drill protocol was then followed to insert the implants. Dental implants were placed in the regenerated alveolar sites of the maxilla or mandible.

### 2.4. Sample Preparation

The specimens underwent decalcification before being embedded in paraffin and sectioned. The samples were fixed in 10% neutral buffered formalin (consisting of 10 mL of 37% formaldehyde solution, 0.8 g of NaCl, 0.4 g of monobasic potassium phosphate, 0.65 g of dibasic potassium phosphate, and 90 mL of distilled water) for a period of 7 days. Decalcification was achieved using disodium EDTA at pH 7, and the process continued until complete decalcification, which was verified through physical assessment. Afterward, the specimens were dehydrated using ethanol solutions of increasing concentrations (70% to 100%), cleared with xylene, and then embedded in paraffin. All chemicals were supplied by Carlo Erba reagents (Dassault Systèmes Group, Vélizy-Villacoublay, France). The paraffin-embedded sections were cut at 5 μm thick using a Leica RM2245 rotary microtome (Leica Biosystems, Nussloch, Germany) and mounted on Superfrost glass slides with Biomount HM bio-optica (Bio-Optica Milano S.p.A., Milan, Italy). The histological sections were stained with Hematoxylin and Eosin (H&E) (Bio-Optica Milano S.p.A., Milan, Italy). The histological images were captured with a transmitted light microscope (Olympus Corporation, Tokyo, Japan) and digitized using a digital camera for analysis via IAS 2000 image analysis software 2024 (QEA) (DeltaPix, Smørum, Denmark). Through histomorphometric analysis, the following measurements were distinguished:

- BV%: the percentage of mineralized tissue, excluding medullary tissues.
- TT%: the percentage of volume occupied by residual graft material, primarily dentin.
- VB%: the percentage of vital bone, excluding medullary tissues.

BV% was calculated as the sum of TT% and VB%, with all measurements conducted using the ImageJ software 1.54d program (National Institutes of Health (NIH), Bethesda, MD, USA).

### 2.5. Statistical Analysis

Statistical analysis was performed to evaluate the clinical and histological outcomes of the alveolar socket preservation procedures. Descriptive statistics were used to summarize the data, including means and standard deviations for continuous variables, and frequencies and percentages for categorical variables.

Comparative analyses between the maxillary and mandibular groups (Group A and Group B) were conducted using Student’s *t*-test for normally distributed variables and the Mann–Whitney U test for non-normally distributed variables.

Categorical data were analyzed using the chi-square test or Fisher’s exact test, depending on the sample size and distribution of the data. The chi-square test was employed when the expected frequency in each category was sufficiently large, ensuring the validity of the test’s assumptions. When the expected cell counts were small, Fisher’s exact test was used to provide a more accurate measure of association between the variables. The statistical significance threshold was set at *p* < 0.05 for all analyses. Data analysis was performed using SPSS software (Version 25.0, IBM Corp., Armonk, NY, USA). Descriptive statistics, including means and standard deviations, were calculated for continuous variables, while frequencies and percentages were reported for categorical data. Additionally, multivariate regression analyses were conducted to adjust for potential confounders, such as age, gender, and defect size, to assess the impact on primary outcomes, such as bone volume and vital bone percentage.

A significance level of 0.05 was set for all statistical tests. Data analysis was performed using SPSS software (Version 25.0, IBM Corp., Armonk, NY, USA). Additionally, multivariate analyses were conducted to adjust for potential confounding factors such as age, gender, and the initial defect size. Regression models were applied to assess the impact of these variables on the primary outcomes of bone volume percentage, vital bone percentage, and residual graft material percentage.

Results were presented with 95% confidence intervals to provide an estimate of the precision of the observed effects. The statistical methods ensured robust analysis and interpretation of the data, allowing for meaningful conclusions regarding the effectiveness of tooth-derived graft materials in alveolar socket preservation procedures.

## 3. Results

A total of 178 patients in good general health were enrolled in the study, resulting in the analysis of 187 post-extractive sockets, which were divided into two groups: Group A (mandibular, 99 samples) and Group B (maxillary, 108 samples) (Table 1).

The mean age of patients in Group A was 58.66 years (±9.97), while in Group B, it was 55.97 years (±11.15). The gender distribution in Group A was 53.54% female and 46.46% male, whereas Group B had a higher proportion of females at 64.81% and 35.19% males.

**Figure 7:**
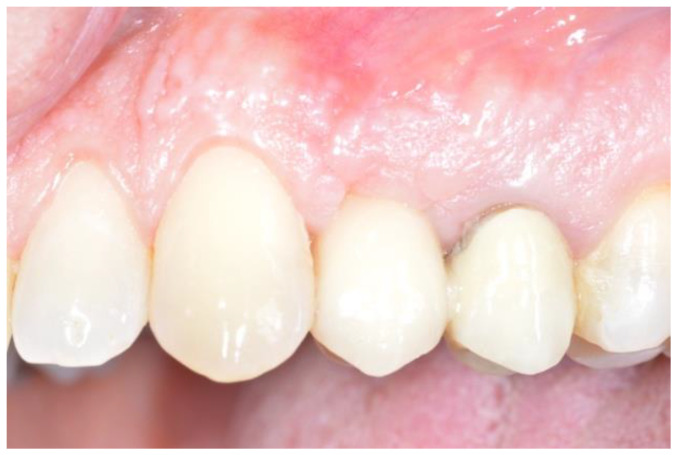
The 2.4 implant 6 months after delivery.

**Figure 8:**
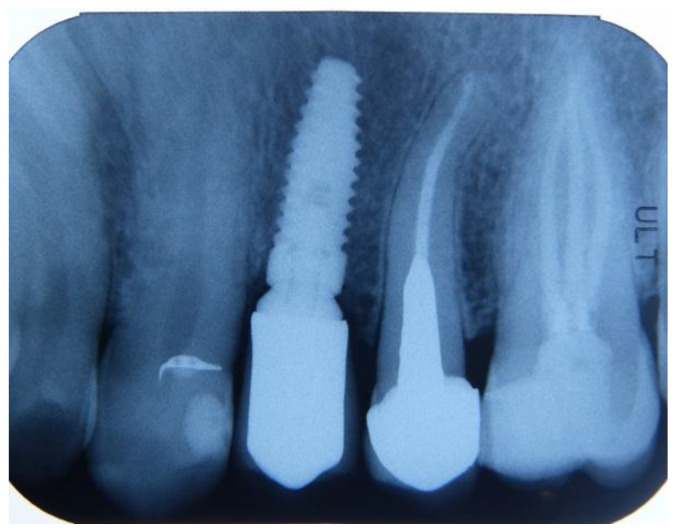
Intraoral radiography of the implant placed on 2.4 site 6 months after delivery.

**Figure 9:**
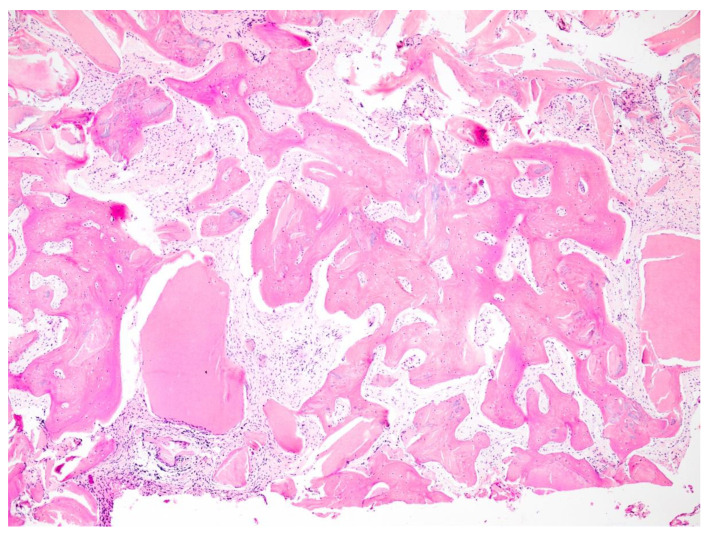
Histological fragment. Total calcified tissue = 50.595%; bone tissue = 48.418%; Tooth Transformer = 2.176%.

All patients demonstrated successful clinical healing of both hard and soft tissues (Figure 7, Figure 8 and Figure 9). In each treated site, a standard titanium implant was successfully inserted after a 5-month healing period. The physiological bone resorption following tooth extraction was minimal, ensuring the preservation of a robust hard tissue architecture. Importantly, no complications, such as suppuration or bleeding, were observed during the healing period. The success rate, defined as the ability to maintain both soft and hard tissues while facilitating implant insertion, was 100% across all cases.

From a histological point of view, the mean bone volume percentage (BV%) was higher in Group A (mandibular) compared to Group B (maxillary) [Figure 9]. Specifically, Group A exhibited a mean BV% of 50.33% (±14.86), while Group B showed a mean BV% of 43.53% (±12.73). Similarly, the mean vital bone percentage (VB%) was greater in Group A at 40.59% (±19.90) compared to 29.70% (±17.68) in Group B. The mean residual graft-Tooth Transformer percentage (TT%) was comparable between the two groups, with Group A at 7.95% (±9.85) and Group B at 6.75% (±9.62).

The regression analysis indicated that only the group variable (maxillary vs mandibular) had a significant impact on vital bone percentage (VB%), with the maxillary group showing a lower VB% compared to the mandibular group. Age and gender did not significantly affect the primary outcomes of bone volume percentage, vital bone percentage, or residual graft material percentage (Table 2).

## 4. Discussion

The present study underscores the effectiveness of autologous dentin grafts, processed using the Tooth Transformer^®^ device, for alveolar socket preservation.

Our data reveal that the dentin graft material produces denser tissue in mandibular sites, which are generally denser, and less dense tissue in maxillary sites, which are usually less dense. This observation supports earlier studies indicating that autologous tooth-derived grafts provide the long-term preservation of bone volume and maintain structural integrity over extended period [15]. The use of autologous biomaterials, such as those derived from teeth, offers significant advantages, including a reduced risk of rejection and enhanced tissue integration due to their biocompatibility and osteoinductive properties [16]. These grafts also eliminate the need for additional surgical sites, which is a considerable benefit for patient comfort and overall procedural efficiency [16].

The Tooth Transformer^®^ biomaterial, in particular, is highly effective due to its ability to recruit and differentiate mesenchymal stem cells, thereby accelerating the regeneration of damaged bone tissues [17]. Furthermore, the role of oral microbiota in promoting bone regeneration and preventing complications, such as surgical site infections, is increasingly recognized. Maintaining an optimal microbiotic balance is crucial for successful outcomes in bone grafting procedures. This aspect adds another layer of benefit to using autologous dentin grafts processed with the Tooth Transformer^®^ device, as they likely support a healthy microbial environment [18].

In a clinical context, the use of the Tooth Transformer^®^ device in multiple international clinics resulted in high success rates for dental implants placed in maxillary sites following alveolar socket preservation procedures. These procedures consistently showed high percentages of bone volume and vital new bone formation, with minimal postoperative complications. This is consistent with findings from other studies which report high implant survival rates and low complication rates when using autologous tooth-derived grafts [9,10,11,12].

The histomorphometric analysis in our study demonstrated that new bone formation varied slightly between the maxilla and mandible, reflecting inherent anatomical and functional differences between these regions. The minimal difference in the percentage of residual graft material suggests that the regeneration process is efficient across both types of bone tissue. This aligns with the existing literature which indicates that while bone density and regeneration rates can vary, the overall efficacy of the graft material remains robust [19].

The differences in osteogenic responses between the maxilla and mandible are significant and are influenced by several factors, including bone density, mechanical loading, and cellular composition. The mandible is denser and experiences higher mechanical loading, which can contribute to a stronger osteogenic response. This greater mechanical stress leads to an increased activation of osteoblasts and osteoclasts, enhancing bone remodeling and regeneration. The mandible’s higher mineral density compared to the maxilla, as demonstrated in several studies, suggests that it may be better equipped to handle functional loads, supporting more robust bone formation under stress [20,21].

On the other hand, the maxilla has a more porous structure with a higher proportion of trabecular bone, which allows for faster vascularization and healing but also greater resorption. The maxilla’s structure, being less dense and more trabecular, promotes quicker bone turnover but may result in less long-term stability compared to the denser mandibular bone [22].

Studies have also shown that bone marrow-derived stem cells from the mandible exhibit a higher osteogenic potential compared to other bones, including the maxilla, highlighting the intrinsic differences in bone regeneration capabilities [21,23]. This distinction is critical in clinical practice, as treatments for bone defects may need to be tailored to the specific anatomical characteristics of each region [22,24].

Thus, the mandible’s denser bone and mechanical loading play a key role in fostering a stronger osteogenic response, while the maxilla’s rapid vascularization contributes to faster, though less stable, bone regeneration.

Many studies [25,26] on the Tooth Transformer were conducted by the Italian group led by Elio Minetti, who is the first author of this article. Other systems have also been proposed. Two articles have compared the BonMaker (Korea Dental Solution Co. Ltd, Busan, South Korea) Tooth Transformer (Tooth Transformer SRL, Milan, Italy), and Smart Dentin Grinder systems (KometaBio, Fort Lee, NJ, USA).

The studies [25,26], including the analysis of BonMaker, Tooth Transformer, and Smart Dentin Grinder, have demonstrated effective bone augmentation and successful osseointegration. Autogenous tooth-derived grafts provided consistent bone regeneration outcomes, confirmed via clinical, morphological, and biochemical evaluations. Minimal non-remodeled material was observed, indicating the material’s high efficiency in facilitating bone formation. Despite structural differences in dentin particles processed using various devices, the clinical results were comparable, supporting the use of patients’ teeth as a reliable source for bone grafts. These findings, validated by histological evidence, suggest that tooth-derived graft materials are a promising alternative in regenerative dentistry.

Another notable study [17] compared the effectiveness of autogenous tooth materials with other bone grafts, particularly xenogeneic substitutes. The study highlighted that autogenous tooth bone grafts showed superior osteoconduction and osteoinduction properties compared to xenogeneic grafts. Moreover, the use of autogenous materials eliminated the need for additional surgical sites, reducing patient discomfort and procedural complexity. The findings from these trials support the use of autogenous dentin grafts as a viable alternative to traditional bone graft materials.

The consistent findings across various studies underscore the biocompatibility, osteoinductive properties, and clinical success of using tooth-derived graft materials [27,28]. As research continues to evolve, the application of autologous dentin grafts in dental and maxillofacial surgery is likely to expand, offering improved outcomes and enhanced patient care [29].

In conclusion, autologous dentin grafts, particularly those processed using the Tooth Transformer, appear to be a promising and biocompatible alternative to allogeneic and synthetic bone substitutes for alveolar socket preservation [30]. The positive clinical outcomes, including high implant survival rates and substantial bone regeneration, support the potential of this approach as a viable option for enhancing bone structure and function in dental procedures [31]. Further research should continue to explore the factors contributing to the observed differences in treatment outcomes between the maxilla and mandible, and the broader applications of autologous tooth-derived graft materials in regenerative dentistry [32].

Additionally, the process of demineralization, a critical step in preparing the tooth material, enhances the release of growth factors such as BMP-2, which are crucial for bone regeneration [33]. This process ensures that the graft material not only serves as a scaffold but also actively promotes new bone formation [34]. Studies have shown that the incorporation of these growth factors can significantly accelerate the healing process and improve the quality of the regenerated bone [35].

Future research should aim to explore the molecular mechanisms underlying the observed differences in bone regeneration between the maxilla and mandible [36]. Understanding these mechanisms could lead to optimized treatment protocols tailored to the specific characteristics of different bone types. Additionally, long-term studies are needed to assess the durability and stability of the regenerated bone over extended periods, which would provide deeper insights into the long-term benefits and potential challenges of using autologous dentin grafts [37].

In summary, the findings of this study, supported by the extensive literature, confirm that autologous dentin grafts processed with the Tooth Transformer^®^ are a viable and effective option for alveolar socket preservation [38]. This method not only offers high biocompatibility and osteoinductive properties, but also aligns with patient-centric approaches in regenerative dentistry, promising enhanced outcomes with fewer complications [39]. As the field of regenerative dentistry continues to evolve, autologous dentin grafts represent a significant advancement, offering a promising alternative to traditional bone graft materials [40]. Further research will undoubtedly continue to refine and expand the applications of this innovative approach, ultimately improving patient care and clinical outcomes in dental and maxillofacial surgery [41].

## Figures and Tables

**Table 1:** Hystomorphometric results.

Graft Site	Number of Samples	Gender Distribution (% F/M)	Bone Volume% (BV%)	Vital Bone% (VB%)	Residual Graft% (TT%)
**Mandibular**	99	53.54%, 46.46%	50.33% (±14.86)	40.59% (±19.90)	7.95% (±9.85)
**Maxillary**	108	64.81%, 35.19%	43.53% (±12.73)	29.70% (±17.68)	6.75% (±9.62)

**Table 2:** Statistical analysis.

Analysis Type	Outcome Measure	Test Statistic	*p*-Value	Significant (*p* < 0.05)
**Comparative Analyses**				
	Bone Volume% (BV%)	t = 3.52	0.0005	Yes
	Vital Bone% (VB%)	t = 4.15	<0.0001	Yes
	Residual Graft-Tooth Transformer%	t = 0.89	376	No
	Bone Volume% (BV%)	U = 6752	0.0011	Yes
	Vital Bone% (VB%)	U = 6853	0.0005	Yes
	Residual Graft-Tooth Transformer%	U = 5664	461	No
	Gender Distribution	Χ^2^ = 2.28	131	No
**Multivariate Analyses**				
	Bone Volume% (BV%)—Age	β = −0.05	395	No
	Bone Volume% (BV%)—Group	β = −1.32	346	No
	Bone Volume% (BV%)—Gender	β = −1.11	424	No
	Vital Bone% (VB%)—Age	β = −0.10	54	No
	Vital Bone% (VB%)—Group	β = −2.67	17	Yes
	Vital Bone% (VB%)—Gender	β = −1.64	145	No
	Residual Graft-Tooth Transformer%—Age	β = −0.05	357	No
	Residual Graft-Tooth Transformer%—Group	β = −1.16	397	No
	Residual Graft-Tooth Transformer%—Gender	β = −1.06	448	No

## Data Availability

The data presented in this study are available on request from the corresponding author due to privacy and legal reasons.
